# Commentary: From the Editor's desk

**DOI:** 10.1155/S1463924681000011

**Published:** 1981

**Authors:** Peter B. Stockwell


### Commentary

### From the Editor's desk

At the commencement of the third volume of the **Journal of Automatic Chemistry**, it is worthwhile to review progress, both in the context of the journal itself, and within automatic chemistry. On the former, we have established a very steady input of papers which augers well for the future. Subscriptions are increasing; however, plainly, the journal has not established its presence in some corners of the market. As with any journal of this nature, there is a necessary requirement to attract advertising revenue, and whilst the level established provides a viable proposition, a more internationally orientated and larger input of advertisements must be aimed for. Given additional revenue from this source, a more frequent publication schedule can be aimed for. Although a quarterly frequency of publication for the third edition is being maintained, a larger issue size will be promoted, so that the time between acceptance of a paper and its publication will be kept to a reasonable minimum. It is important to balance each issue between clinical and industrial interests, and not to place undue emphasis on instrument evaluations; the increased issue size will help in this respect.

### Multidisciplinary approaches

It has often been stated in these columns that the subject of automated chemistry, be it for the industrial or for the clinical market, requires a multidisciplinary approach. Chemistry must be interfaced with electronics, mechanical engineering, computer techniques and software developments, amongst other aspects. In addition, a proper protocol must be established in order to obtain data which can be correlated, both within a laboratory, and between laboratories.

It is promising that over the last year in the UK two significant developments have been made which will promote the concepts of a multidisciplinary education, and also will establish closer links between academia and industry. A new department of Instrumentation and Analytical Science has been established at the University of Manchester Institute of Science and Technology (UMIST), and at University College Swansea a personal chair of Analytical Sciences has been set up.

The Department of Instrumentation and Analytical Science at UMIST seeks to unify instrumentation and analytical sciences as a discipline capable of being taught as a single integrated unit at the highest academic and professional level. Many Institutions teach analytical chemistry directed towards the training of analysts. The role of this new department is primarily that of imparting to scientists and technologists, from a wide range of disciplines, an understanding of the principles and practice of measurement in its broadest sense. It will train specialists whose professional role would be initially that associated with the design of instrumentation and the development of new analytical techniques. UMIST has an excellent record of collaborative work which crosses traditional academic boundaries and frequently involves industrial contacts. This pattern will provide a model for the future activities of the new department.

Dr. Gordon F. Kirkbright has been appointed Professor of Analytical Sciences and Head of the Department; his background is in analytical chemistry. Two other chairs have been established to provide the multidisciplinary co-operation. Peter Payne, whose background is in bio-engineering, has been appointed to a Chair of Instrumentation; and Maurice Beck has similarly been appointed; his particular interests are in control engineering. It is therefore clear that a broad spectrum of measurement activities, both in research and teaching, will develop rapidly at UMIST. Already, in early 1981, the total staff complement of the department is 35 and expected to double within the next year.

The approach by the University College of Swansea, whilst not being as bold as that at UMIST, is none the less significant. Professor D. Betteridge has been established in a personal chair of analytical sciences. Degree programmes in analytical science have been established, and plans made to set up postgraduate courses. Short term concentrated teaching programmes, as illustrated by the Summer School of Automatic Chemical Analysis, will form a major area of activity for this group. Again, strong ties between senior industrial researchers will be promoted, as well as co-operation from academic colleagues in other departments to work on multidisciplinary projects, particularly those involving microelectronics. These activities will serve to augment the already established programme of research in automated analytical chemistry pioneered by Prof. Betteridge and his group.

Both groups should be encouraged and I am sure from their beginnings that they will shortly have a valuable input in the sphere of automated analytical chemistry.

### Highlights of 1980

It is almost impossible to cover all the developments made over the last year in a short summary, and any attempt to do so would not be rewarding. However, a few aspects appear to me to be particularly significant. After what can only be considered a very long gestation period, the solid phase, or layer chemistry, developed by Eastman Kodak has been launched on the clinical market.

In addition to the reflectance spectroscopy used for glucose measurements etc., an electrolyte version has also been developed. There has been a similar advancement from the Ames group in their Seralyzer developments. Both of these approaches will obviously benefit from the ground work carried out by the DuPont company when establishing a market for their ACA equipment. All of these approaches take a great deal of the responsibility for method developments and research away from clinical chemists and place considerable responsibility upon the instrument companies. The problems inherent in this approach have been discussed by Bierens de Haan in this journal. A further exciting development, this time in flow injection analysis, was described by the Beltsville Group at the Pittsburgh Conference 1980. The FIA technique is used to carry out titrations and only a timed response is measured which is both simple and accurate. This group has also made significant advances in developing the theory of FIA.

Many developments have been made throughout 1980 using microprocessor technologies. Of these, one instrument developed by Analytical Instruments of Cambridge UK deserves special mention. Their gas chromatograph, the A1-90, represents a considerable advance on those available on the market and provides a unique approach which integrates a graphics terminal onto a broad range of chomatographic packages and detectors. It is refreshing to see a small UK company making such a significant impact on the market.

*Peter B. Stockwell*

